# A refashioned foley catheter: novel cost-effective available stent in pediatric laryngotracheal reconstruction

**DOI:** 10.1007/s00405-022-07707-x

**Published:** 2022-10-25

**Authors:** Ahmed El-Sobki, Mohamed Elkahwagi, Mohamed E. El-Deeb, Fedaey R. Habaza, Mohammed Abdelbadie Salem, Ahmed Hemdan, Noha Ahmed El-Kholy, Mahmoud Elsaid Ibrahim Alsobky

**Affiliations:** 1 Otorhinolaryngology Department, Faculty of Medicine, Mansoura University, Mansoura, Egypt; 2 Otorhinolaryngology Department, Faculty of Medicine, Kafrelsheikh University, El-Giesh Street, Kafrelsheikh, Egypt; 3 Otorhinolaryngology Department, Faculty of Medicine, Al-Azhar University Damietta, Al Hayy Ath Thani, Egypt

**Keywords:** Laryngotracheal, Stenosis, Reconstruction, Stent, Stenting

## Abstract

**Purpose:**

Pediatric laryngotracheal reconstruction (LTR) for laryngotracheal stenosis (LTS) mandates stenting in certain situations. This study presents a novel commercially available and cost-effective stent, a refashioned foley catheter.

**Methods:**

This prospective clinical study was performed on pediatric cases with LTS up to 8 years. The study was performed in a tertiary referral center. The selection of the proper foley catheter size for age was explained. The atraumatic insertion maneuver of the stent was also shown in detail in different situations of LTS. The endoscopic removal of the stent was also described. The mean follow-up was 6.45 ± 1.3 months.

**Results:**

The study included 31 cases using the refashioned foley catheter stents. The study included 17 males and 14 females with a mean age of 3.45 ± 1.09. Subglottic stenosis was the most common cause of LTR in the study (74.2%) cases. The mean duration of stenting was 40.5 ± 3.7 days. Decannulation was achieved in 96.8% of cases. No stent complications were encountered like stent migration, excess granulation tissue, intractable aspiration, or pressure necrosis.

**Conclusion:**

The refashioned foley catheter is a novel, available, and inexpensive stent that can be utilized for LTR cases for pediatric LTS. The newly described stent is soft, pliable with atraumatic insertion and easy endoscopic removal with minimal complications.

## Introduction

Pediatric laryngotracheal stenosis (LTS) is a challenging condition to be managed [1]. It can be either congenital or acquired narrowing of the airway, affecting the glottis, subglottis, and/or trachea [2]. In congenital lesions, it may be due to webs formation, as seen in anterior glottic webs, cricoid cartilage maldevelopment, or bilateral vocal fold paralysis [3]. However, 90% of the cases are attributed to acquired LTS, such as intubation injury caused by prolonged intubation, although brief intubation may cause injury in the pediatric population [4, 5]. Combined glottic and subglottic stenosis is linked with delayed and decreased rates of decannulation when compared with isolated subglottic stenosis[6].

The main problems of LTS are airway ones like dyspnea on exertion, stridor, and voice disorder as a weak voice [3]. The surgical standard for repair of LTS has become the laryngotracheal reconstruction (LTR) with stent placement [7]. The use of stents in pediatric laryngotracheal reconstruction (LTR) is a common practice [8]. Stents are frequently used to maintain postsurgical patency and caliber of the airway after LTR and stabilize the airway framework [9]. Timing of stent placement, stent material, stent design, and duration of stenting play a significant role in surgical outcomes for children with LTS [10].

Ideally, stents used after LTR should be rigid enough to keep the reconstructed area and grafts stable in position, allow oral intake of food without aspiration, minimize granulation tissue formation, be easy to examine and remove, and be harmonious with tracheotomy tube cares and changes [11]. Many stents are commercially accessible and intended to improve glottic and subglottic patency after LTR. Still, the majority has a poor representation of laryngeal anatomy, particularly concerning the complex shape of the glottis [12]. In addition, most of them are expensive. Over the last decade, very little progress has been made in the invention of new stents in postoperative LTR patients [1]. Although an ideal supra-stomal stent does not exist, many stents are present, like the soft silicone Montgomery T-tube, the Teflon Aboulker stent, the Eliachar laryngotracheal (LT) stent, and the laryngotracheal mold of Monnier [13, 14].

This study presents a novel, cost-effective and available stent for use in LTR with good support for the laryngeal framework and with suitable accommodation to the complex glottic shape.

## Patients and methods

This clinical study was performed on cases with LTS that were admitted to Mansoura university hospital in the department of ORL–HNS during the period from 2018 to 2021. Institutional review board approval (IRB) was obtained from Mansoura University Hospital's ethical committee with a code of R.20.05.842. Children aged 1–8 years with LTS with airway and voice problems were included. Data of the included patients were collected and included gender, age, type and level of stenosis, and the state of stoma, either tracheostomized or not.

### Surgical technique

Foley catheter (Euro foley catheter, Euromed for medical industries, Nasr City, Cairo, Egypt) was selected as a novel stent in pediatric cases in this study. It was selected for being soft, pliable, and atraumatic as it was originally designed to be in contact with the urethra.

Selection of the suitable size of the catheter was the first important step. First, we selected the inner diameter (ID) of the endotracheal tube (ETT) that matched the child’s age. This was performed utilizing the standard equation for that, which was (age/4) + 4. Each ETT had an ID and outer diameter (OD). The OD was roughly 2 mm larger than the ID. Having a standardized outer diameter (OD) of the foley catheter enabled us to select the catheter size equal to or near the assumed airway size according to the child's age. Table 1 shows the distribution of catheter sizes used for the study population.

**Table 1:** Distribution of catheter sizes used for the study population

French size	Number of cases
16	2
18	3
20	12
22	11
24	3

The French system is a simple one used to measure the size of a catheter, one increment on the French scale is equal to 1/3 mm, and thus 1 mm = 3 Fr, e.g., 12 Fr catheter is 12 × 0.33 mm = 4 mm in caliber.

The length of the foley was adjusted, so that the proximal end lay opposite the ventricle and the distal end 5 mm above the stoma. The proximal end was sutured with a 2/0 proline suture to close the upper end of the lumen to prevent aspiration postoperatively.

After vertically incising the airway in the midline and suturing the posterior graft in situ, if needed, the stent was lubricated with antibiotic ointment. We started with pushing the proximal end of the stent from below upward between the vocal cords to the ventricle level; then, the distal end was squeezed to the distal airway. At this stage, an endoscopic examination to detect the level of the stent proximally was done before suturing, and the endotracheal tube was removed to confirm that the caudal end of the stent was not reaching the stoma.

**Fig. 1:**
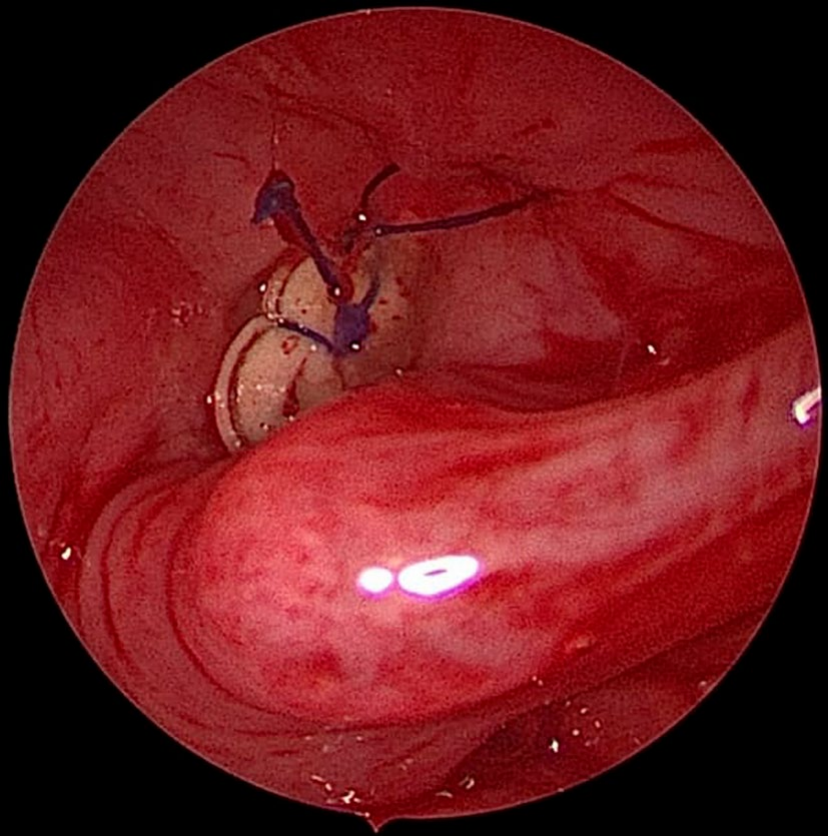
Endoscopic view of the stent with the apex of this stent directed towards the anterior commissure

To prevent aspiration, the upper end of the stent should be closed. We used two proline sutures for this purpose. We strive to give the upper end of the stent a triangular shape with the apex directed toward the anterior commissure, while the base directed toward the posterior commissure. For this purpose, one suture is put towards the future anterior end and the second suture is put in the middle of the upper end of the stent. This is routinely done for all cases with more concern when glottic involvement is there to gain as much sharp anterior commissure as possible (Fig. 1).

**Fig. 2:**
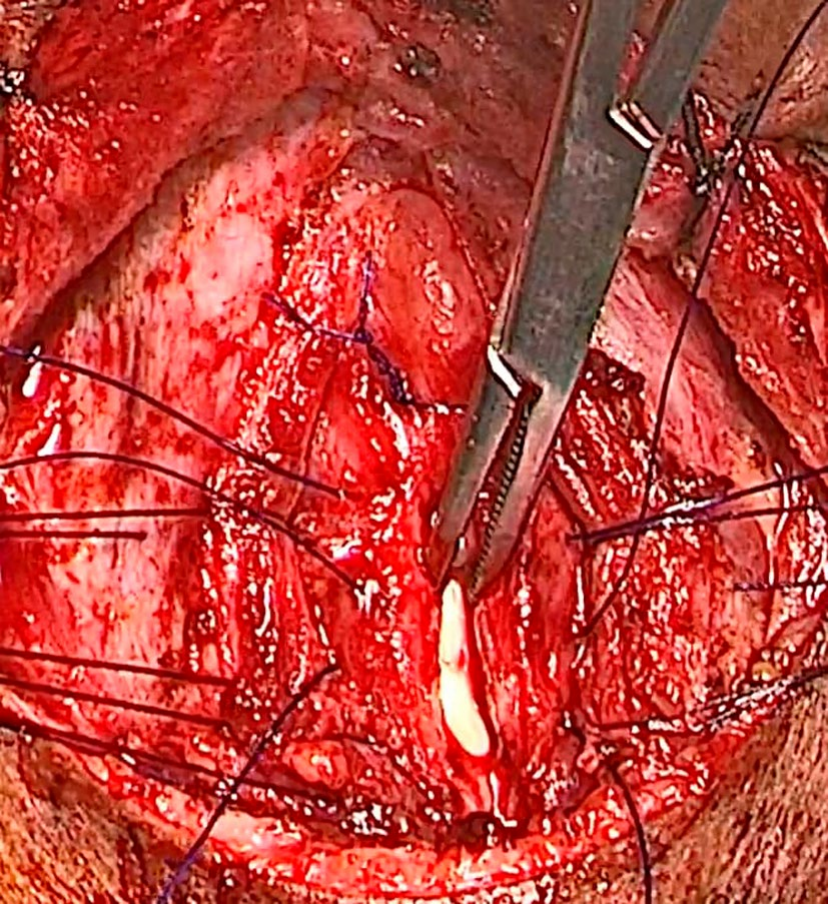
Suturing of the stent to the splitted cricoid cartilage and trachea

To stabilize the stent in place, the proline suture was passed from the splitted cricoid arch, then through the stent and the arch on the contralateral side. An additional suture is passed through the splitted tracheal wall to the stent and then to the wall on the opposite side (Fig. 2).

**Fig. 3:**
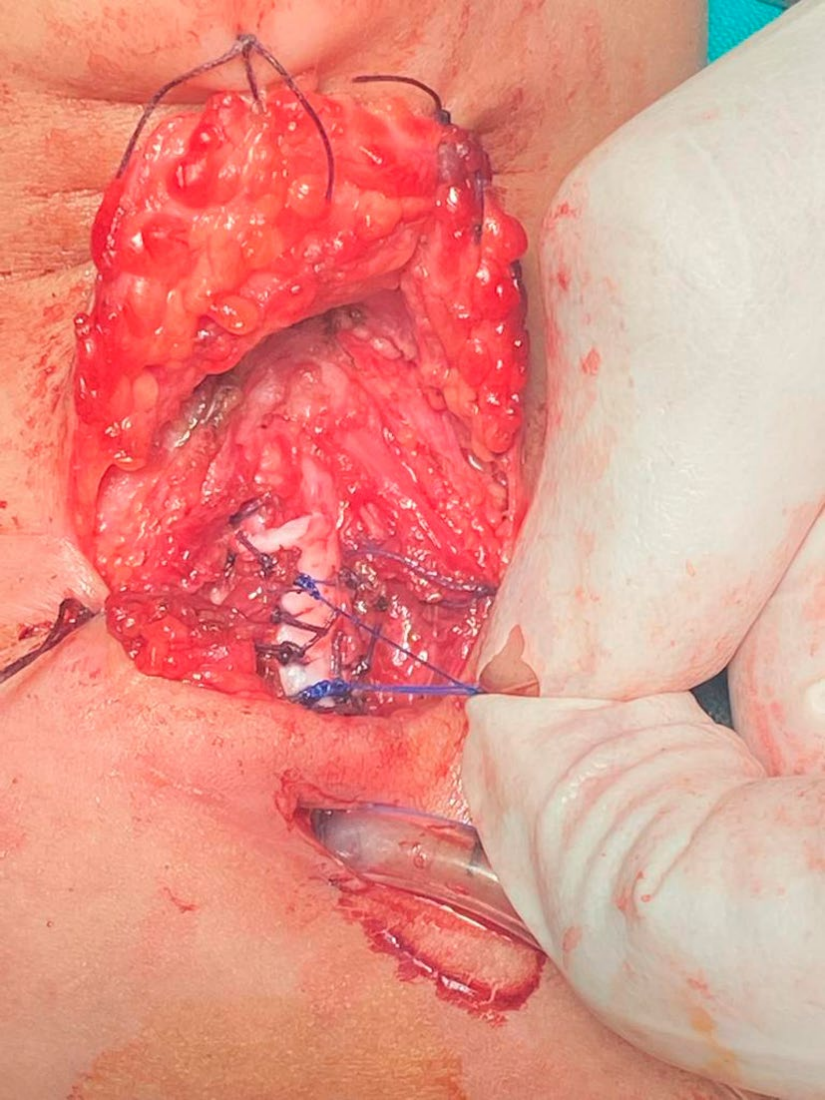
Suturing of the splayed ends of the proline suture and tying over the anterior graft for more stabilization

If an anterior graft was decided, it was sutured in place first, then the splayed ends of the proline suture were tied over the anterior graft providing more anchoring (Fig. 3).

Great care was given to ensure that the sutures were passed through the lumen of the stent, not inside the wall, to make endoscopic stent removal straighter forward.

When surgery mandated a laryngofissure, an additional suture was passed through the ventricles for more anchoring. When the pathology was a congenital thick web, the stent size was selected at the upper limit the lumen might accommodate. The proximal end of the stent was fitted against the anterior commissure trying maximally to have a sharp anterior commissure.

The duration of stenting ranged from 3 to 6 weeks. For stent removal, the larynx was suspended, putting the proximal end of the stent in the endoscopic view. The suture closing the proximal end of the stent was cut with a micro laryngeal scissor giving access to the lumen of the stent. Advancing the endoscope through the lumen of the stent gave access to the proline threads, which were then cut, and the stent was smoothly pulled out**.**

At this stage, granulation tissue was in the cavity, and it disappeared on it is own later on. Still, we routinely filled the cavity with antibiotic steroid ointment for a better outcome.

If the fixing suture had passed through the wall, not the lumen of the stent, and the threads were not identified in the lumen, there would be some difficulty with stent removal, where the scissor has to pass between the stent and the airway wall to cut the suture blindly.

Injection antibiotics were prescribed for ten days postoperatively, followed by oral antibiotics for another ten days to prevent bacterial colonization over the stent and postoperative infection. The follow-up was designed to be daily for the first postoperative week, then weekly till the time for stent removal.

The follow-up was designed to be weekly till stent removal. The patients were admitted after the removal of the stent for decannulation to be under observation. The follow-up was designed to be weekly for the 1st month after decannulation and every 2 weeks for the coming 2 months, then monthly till 6 months after decannulation or airway stabilization.

### Statistical analysis

Qualitative data were described using numbers and percentages. Continuous variables were presented as mean ± standard deviation for parametric data.

## Results

Our study included 31 patients with a mean age of 3.45 ± 1.09 years. In four patients, the underlying pathology was a thick congenital laryngeal web; 23 were diagnosed with SGS, one with congenital bilateral abductor paralysis (CBAP), and three patients had posterior glottis stenosis (PGS). Seven patients (22.58%) had associated comorbidities; two patients had cardiac problems (one case of patent ductus arteriosus and a case of tetralogy of Fallot (TOF)), four patients had associated neurological problems (two cases of cerebral palsy and two cases of epilepsy), and one patient had paraplegia after a motor car accident (Table 2).

**Table 2:** Demographic and clinical characteristics among the studied group

Patients characteristics	Study group (*n* = 31)
Age/years (mean ± SD)	3.45 ± 1.09
Sex	
Male	17 (54.8%)
Female	14 (46.2%)
Comorbidity	
Yes	7 (22.58%)
Patent ductus arteriosus	1 (3.23%)
TOF	1 (3.23%)
Cerebral palsy	2 (6.45%)
Epilepsy	2 (6.45%)
Paraplegia	1 (3.23%)
No	24 (77.42%)
Diagnosis and underlying etiology	
Web (Congenital)	4 (12.9%)
SGS	23 (74.2%)
Congenital	3 (13%)
Post-intubation	20 (87%)
CBAP	1 (3.2%)
PGS	3 (9.7%)
Cotton–Myer grade of individuals with SGS (*N* = 23)	
Grade II	10 (43.48%)
Grade III	13(56.52%)
Prior surgery	
Balloon dilatation	20 (64.5%)
Suture lateralization	1 (3.2%)
Laser web excision	1 (3.2%)

*SGS* subglottic stenosis, *CBAP* congenital bilateral abductor paralysis, *SD* standard deviation, *PGS* posterior glottis stenosis, *TOF* tetralogy of Fallot

All our acquired stenosis cases had at least one trial balloon dilatation before deciding on open reconstruction. One of the web cases had laser excision of the web in another institute without improvement of breathing symptoms and followed by rewebbing; thus, we decided to expand the cricoid simultaneously. Suture lateralization was performed on the paralysis before the tracheostomy but failed to achieve adequate airway.

The frequency of endoscopies needed after each surgery ranged from 2 to 4, while the duration of the stent in our patients was 40.5 ± 3.7 days. Regarding complications, surgical removal of granulation tissue was needed with steroid ointment application in 9 cases. Also, a case of SGS required a revision LTR with stenting for a short graft. One case required surgical excision of suprastomal granuloma (Table 3).

**Table 3:** Postoperative complications and outcome measures

Outcome measures	(*n* = 31)
Stent duration (days)	40.5 ± 3.7
Number of endoscopies needed after each surgery	2.71 ± 0.71
Complications	
Wound infection	2 (6.4%)
Graft loss/prolapse	1 (3.2%)
Granulation tissue	9 (29.03%)
Suprastomal granuloma	1 (3.2%)
Outcome	
Decannulated	30 (96.8%)
Not decannulated	1 (3.2%)
Adjunct procedures required	
Granulation tissue removal + steroid application	9 (29.03%)
Removal of suprastomal granuloma	1 (3.2%)
Revision LTR	1 (3.2%)
Cricotracheal resection and anastomosis	1 (3.2%)
Laser treatment and keel insertion	1 (3.2%)

*LTR* laryngotracheal reconstruction

After including the revision LTR case, 30 out of our patients were decannulated at the end of the present study. The remaining case was successfully decannulated after partial cricotracheal resection (PCTR). One case had a residual anterior webbing which necessitated laser treatment and keel insertion.

No stent complications have been detected in this study, like migration, intractable aspiration, or severe postoperative infection.

## Discussion

Pediatric LTS is more challenging than adults due to the smaller pediatric airway, softer and easily collapsible laryngeal cartilages, more complex glottic configuration, and less desaturation tolerance. LTR with stent placement is definitive management for cases with LTS [15]. Stents are usually used to stabilize the laryngeal framework and maintain airway patency after LTR [16]. Stent placement in cases of LTR has been significantly studied in the literature with many commercially available stents and many modifications [17]. The cost and the availability of most of these stents are essential issues that could hinder many surgeons from performing the ideal work. This study provides a novel stent that is commercially available and inexpensive. The stent utilized in the study is a refashioned foley catheter.

The ideal stent to be used in cases of LTR should have the ability to fit the complex configuration of the larynx, especially the glottis [11]. It should be pliable, soft, and easy to be inserted. It should be formed of an inert material so as not to cause reactions and excessive granulation tissue formation [1]. In addition, it should be able to remain in place with minimal migration ability. It should be commercially available and present in many sizes to be accommodated in various airway sizes. In addition, it has to be easily inserted and easily removed [3]. The Teflon Aboulker stent has many of these criteria [8]. However, granulation tissue is formed between it and the stoma in cases of a short stent [11]. In addition, the modified long-fenestrated Aboulker stent carried problems of stent fracture and tracheostomy problems [1]. The Montgomery T-tube stent is made of softer silicone [14]. However, its utility in children, especially younger than 4 years, has been doubted due to the problems of mucous plugging of the lumen. In addition, it cannot be relied on in cases of SGS with posterior glottic stenosis [12]. The Eliachar laryngotracheal (LT) stent, made of silicone rubber which is less traumatic, is also available, but it has two adult sizes only. Despite having a good conformation to the endolaryngeal anatomy, its shape is not triangular at the level of the glottis, and it neither restores a large interarytenoid distance nor produces a sharp anterior commissure [13].

The Rutter stents are soft and deformable with rounded and smooth distal ends while the proximal end is trimmed to the level of the false vocal cords before being plugged with a rounded cap that is less likely to induce epiglottic granulation tissue [18]. They are used in the USA with good results, but unavailability in our institutes hinders their use.

The foley catheter utilized in this study has many of the ideal features. It is a soft atraumatic stent made of inert material, and its application and removal are not complicated. In addition, it is commercially available in many sizes that are suitable for all pediatric ages up to 8 years. Therefore, we utilized this refashioned catheter for that purpose and achieved comparable results to the studies that utilized other types of stents.

Before using foley catheters, we used the vertical limb Montgomery T tubes with the disadvantage of high cost and limited availability, silicon urinary catheters, and custom-made silicone stents with the disadvantage of excessive granulation tissue.

The most common advantage of our stent, besides its availability, is its low cost. It usually costs only 1–2 USD compared to the Montgomery tube, which costs around 300 USD.

As for the stenting duration, it was never less than three weeks, which seems sufficient to support the graft until it starts to be incorporated within the framework. However, for cases where anterior and posterior grafts were inserted, we need to keep the framework stabilized, or where a glottic web was simultaneously cut with cricoid expansion, where rewebbing needs to be prevented, and a longer duration of stenting is usually needed. Smith et al. [19] identify several advantages of using long-term stenting, they showed that a higher proportion of children with long-term stenting were successfully decannulated. Long-term stenting improved outcomes for children with SGS undergoing LTR in their population.

Postoperative aspiration is a problem that could occur with small stents or poorly designed stents in the glottic region [20]. This condition can affect the outcome due to recurrent infection over the repair site and earlier stent removal [11]. In our study, no intractable postoperative aspiration was detected. This can be attributed to the proper size of the foley catheter that matches the airway size for the age. In addition, suturing of the upper end of the stent prevented fluid from leaking through its lumen. In addition, the pliable nature of the catheter enabled the stent to accommodate the complex glottic configuration with an atraumatic insertion maneuver.

Excessive healing and granulation tissue formation is another postoperative complication in cases of LTR, especially with stent application [20]. Postoperative bacterial and fungal colonization is a stated condition that occurs over the stent material [21]. This condition can promote excessive granulation tissue formation that can impair the repair site or encroach the airway lumen [10]. Therefore, the postoperative antibiotic cover is an essential matter in stenting cases [21]. In addition, the configuration of the stent affects the process of granulation tissue formation [12]. Zalzal et al. described the excessive granulation and pressure necrosis with the Aboulker stent despite being formed of polished inert Teflon [8]. Later on, this stent was modified by adding a round-shaped cap to the distal end of the stent [12]. In our study, minimal granulation tissue was encountered in the airway following stent removal, and on further endoscopic airway examination, this granulation tissue disappeared. This can be attributed to the soft and pliable nature of the foley and the that it is an inert material that does not promote the excessive reaction. In addition, the antibiotic ointment was utilized to cover the stent on application and the airway lumen after stent removal. All these factors make the foley catheter an ideal stent with minimal reaction.

In our study, there was a case of suprastomal granulation formation requiring removal. This is a known occurrence with tracheostomy tubes as it develops from chronic frictional trauma from the tracheotomy tube and suprastomal stents, especially when they fall short of the stoma [22]. Schweiger et al. presented two cases of inverting suprastomal granulomas that reached the tip of the patient's tracheotomy tube or beyond. Large granulomas with tracheal extension, unlike the majority of suprastomal granulomas, are likely to be symptomatic and require open excision [23].

One limitation of the study is the age limit, which we limited up to 8 years with foley catheter 24 as this is the largest available one. In addition, older children may need a more rigid type of stent. Another limitation of our work is the lack of direct comparison with other available stents. Future multicentric studies are needed to evaluate the efficacy of the foley catheter for use in LTR and to validate its role.

## Conclusion

The novel refashioned foley catheter can be utilized for stenting in LTR performed for pediatric LTS with good postoperative results. It can be considered the most commercially available and cost-effective stent ever present in the literature.

## Data Availability

The data sets used and/or analyzed during the current study are available from the corresponding author on reasonable request.
